# Perceptions on violence against women and its impacts on mental health and response mechanisms among community-based stakeholders: a qualitative study from Nepal

**DOI:** 10.1186/s12905-024-03064-5

**Published:** 2024-04-24

**Authors:** Rachana Shrestha, Diksha Sapkota, Raunak Raj Sarraf, Devika Mehra, Anna Mia Ekström, Keshab Deuba

**Affiliations:** 1 Public Health and Environment Research Centre (PERC), Lalitpur, Nepal; 2 Knowledge to Action (K2A), Lalitpur, Nepal; 3 Griffith Criminology Institute, Queensland, Australia; 4 Sano Paila, Birgunj, Nepal; 5 MAMTA Health Institute for Mother and Child, New Delhi, India; 6 Public Health Consultant, Medeon Science Park, Malmö, Sweden; 7 Department of Global Public Health, Karolinska Institutet, Stockholm, Sweden; 8 Department of Infectious Diseases, South General Hospital, Stockholm, Sweden

**Keywords:** Health care providers, Nepal, Perceptions, Qualitative study, Violence against women

## Abstract

**Background:**

Violence against women (VAW) is a significant public health problem. With the emergence of the COVID-19 pandemic, the frequency and severity of VAW has escalated globally. Approximately one in four women in Nepal have been exposed to either physical, psychological, and/or sexual violence in their lifetime, with husbands or male partners being the perpetrators in most cases. VAW prevention has been under-researched in low- and middle-income countries, including Nepal. This study aims to explore the perspectives of local stakeholders, including healthcare providers and survivors of violence in Madhesh Province. The overarching goal is to provide insights for designing prevention and support programs that are acceptable to communities and cater to the needs of survivors.

**Methods:**

An explorative qualitative study was conducted in Madhesh Province, southern Nepal. A total of 21 interviews, including 15 in-depth interviews (IDIs) with health care providers, three IDIs with women seeking general or maternal and child health services at health care centres, three key informant interviews with the local stakeholders working in the field of VAW, and one focus group discussion with violence survivors, were conducted in Nepali by trained field interviewers. Interviews were recorded, transcribed, translated into English, and analysed using content analysis.

**Results:**

VAW, particularly physical violence, was a common experience in the study area. Sociocultural traditions such as dowry, child marriages and son preference were identifiable triggers for VAW, causing significant physical injuries and mental health problems, including suicide. Health care providers reported that violence survivors often hide their experiences of violence and do not seek any kind of help. Women feared that violence would increase in frequency and intensity if their perpetrators found out that they had disclosed their experiences of violence to health care providers. Local stakeholders emphasized the importance of engaging community leaders and garnering support from both women and men in interventions designed to reduce VAW and its impacts on mental health.

**Conclusions:**

Participants reported that verbal and physical violence is often perceived as a normal part of women’s lives. Women should be made aware of available support services and empowered and supported to increase access and uptake of these services. Additionally, more individual-based counselling sessions that encourage women to escape violence and its mental health consequences while maintaining privacy and confidentiality are recommended.

## Background

Violence against women (VAW) is a significant global public health problem, affecting one in three women at least once in their lifetime [1, 2]. VAW has both immediate and long-term consequences, including severe physical injuries that can sometimes result in death, sexual and reproductive problems, and mental illness sometimes leading to suicide [1, 2]. Additionally, VAW also has detrimental effects on women’s education, employment, freedom of choice, and economic empowerment. The causes of violence are multidimensional, encompassing social, economic, cultural, political, and religious reasons, spanning across institutional, community, and individual levels. Low levels of education, exposure to or witnessing to violence during childhood, male privilege, and women’s subordinate status within families and communities are associated with both perpetration and the risk of experiencing or staying in a violent relationship [1]. In this paper, intimate partner violence will be used to refer to any form of violence perpetrated against women by their husbands or male partners [1].

In Nepal, several cultural practices that continue to undermine women’s decision-making power and status, and increase the vulnerability of children’s and young women to violence are banned by law. These practices include child marriage, *deuki* (where daughters are offered to the temple for good fortune), *kamlari* (a bonded labor system where girls must work at their landowner’s house) [3] and dowry, which is still common in the southern part of Nepal [4]. However, most cases of dowry-related violence go unreported and are resolved outside of the court. Brides whose families are unable to meet dowry demands often face various forms of VAW. Reported physical violence includes beatings, burnings, hangings, poisonings, and torture, resulting in suicide or apparent suicide, and murder. These traditional practices persist because women and girls are sometimes viewed merely as objects or symbols rather than as human beings of equal value [5].

A national survey in Nepal estimated that 26% of women have experienced violence at least once in their lifetime [6], and the prevalence is even higher in Madhesh Province, the southeastern part of Nepal bordering with India. More than two-thirds of VAW survivors in Nepal never seek help, primarily due to pre-existing patriarchal social norms that normalize violence as a part of married life, and/or due to fear and social stigma are associated with seeking support services for VAW [6–8].

Child marriage (marriage before the age of 20) has been illegal in Nepal since 1963, and the government has endorsed a National Strategy to End Child Marriage in Nepal by 2030. However, child marriage is still commonly practiced, particularly in rural areas, without proper legal enforcement. Only 400 cases of child marriage were reported to the police from 1996 to 2018 [4], which is extremely low considering that around one third of Nepalese girls are married before the age of 18 [9].

A Multiple Indicator Cluster Survey from Nepal found that 8% of women aged 20 to 24 years had been married before the age of 15 and 33% before age 18 [9]. The proportion of early or child marriage was highest in Madhesh Province, where 14% were married before the age of 15 and 46% before their 18th birthday [9]. The main driver of child marriage is poverty, as marrying off daughters early means fewer mouths to feed [4] and families also pay higher dowries for older and educated girls [10]. This leads to poor families attempting to marry their daughters at a young age. Furthermore, there is a stigma surrounding what is perceived as “late marriages” (after the age of 20 in rural settings and after the age of 25 in urban settings), which may undermine family honour. Therefore, marrying girls before their sexual debut and at a very young age is seen as an advantage [11]. A literature review also found that son preference and discrimination against girls within the family also influence the age of marriage [4].

Health care providers often serve as the first point of contact and could play an essential role in addressing the needs of violence survivors who frequently visit health care centers for physical and psychological symptoms without disclosing their history of violence [12, 13]. The government has launched a hospital-based integrated support initiative called “One-Stop Crisis Management Centres”, for women seeking care due to VAW. Another government initiative, “*Beti Bachao, Beti Padhao*” (Save the Daughter, Educate the Daughter) [14], is an insurance scheme aimed at empowering and protecting girl children against child marriage and dowry [15], both significant risk factors for VAW.

Previous qualitative research on VAW in Nepal [8, 16–18] lacks empirical evidence on effective interventions, although it has been shown that involving local resources may be effective for preventing VAW [19]. Therefore, it is important to contextualize interventions and explore the perspectives and awareness of VAW among local stakeholders. Involving VAW survivors, health care providers, and local stakeholders in program design is critical for ensuring success and sustainability, but this is rarely considered in practice or documented in the literature [19].

There is very limited evidence on how local communities and stakeholders in Nepalese societies perceive VAW, its causes, impacts, and potential preventive interventions. The current study aims to address this knowledge gap by exploring the perspectives of local stakeholders, health care providers, and survivors of VAW in Madhesh Province, one of the regions most severely affected by VAW in Nepal. The overarching goal is to provide new insights for the design of prevention and support programs that are acceptable to communities, address survivors’ needs, and learn from previous strategies that have shown limited effectiveness [19].

## Methods

### Study design

This study employed a qualitative approach to understand the community perceptions regarding VAW, with a particular focus on the causes, consequences, shortcomings in existing measures against VAW, and suggestions for a future intervention or support programs. This knowledge would provide useful insights into the development and evaluation of an integrated intervention for violence victims in Nepal.

### Study setting

The study was conducted at health care facilities and local non-governmental organizations (NGOs). Twenty-four health care facilities, including district hospitals and primary health centres located across eight districts in Madhesh Province, were randomly selected. Our sampling approach was based on a linked study in which we utilized a random selection process to choose 24 public health facilities from a comprehensive list of public hospitals and primary health care centers in the Madhesh Province. The objective of this linked study was to implement interventions aimed at addressing mental health issues and providing secondary prevention of violence for women who visit these health facilities. Further details related to this linked study are available at the following link: https://www.researchprotocols.org/2023/1/e45917/.

For the current formative assessment, we visited the selected public health facilities for the aforementioned purposes and conducted interviews with health workers and women who were visiting these facilities. Despite our attempts to interview all 24 selected health workers, we encountered difficulties in conducting interviews with nine of them due to various reasons. Among these, three health workers were heavily occupied with their duties at the health facility, two were attending training sessions, one was on leave, and three were engaged in a one-day immunization program at the community level. The health facilities from which we were unable to interview health workers include Simara Pipara Primary Health Center, Jamuniya Primary Health Center, Rajpur Faradwa Primary Health Center, Janakpur Hospital, Yadhukuwa Primary Health Center, Kanchanpur Primary Health Center, Topa Primary Health Center, Kadarbona Primary Health Center, and Kalyanpur Primary Health Center. Additionally, we collaborated with a local partner (Sano Paila) who works in the field of violence against women to interview key stakeholders, such as representatives from NGOs and community women, inorder to incorporate their views and perceptions related to VAW and the available support mechanisms.

Eligible participants available during the visit were approached for ethical consent and recruitment. Although this province has various levels of health facilities, our study only included study participants (health care providers and women seeking general health or maternal and child health services) from provincial and district hospitals and primary health care centres. These facilities constitute a large proportion of health facilities in Nepal and offer a wide range of healthcare services to the public. Additionally, these services are more advanced compared to those provided at basic health posts. One-stop crisis management centers that provide integrated services to survivors of violence are available in a limited number of hospitals at the district level or higher. These dedicated health facilities provide a platform for women visiting the centers and health care providers to share their experiences regarding the available measures to address violence against women. By including such public health facilities in our study, we aim to enhance understanding of the effectiveness of existing interventions in the current context.

### Study participants

The study participants consisted of health care providers, including nurses and auxiliary nurse midwives, women seeking general health or maternal and child health services at health care centers, women with a history of violence, and individuals working in local community-based organizations addressing VAW (see Table 1 for details). The study investigators, in close coordination with Sano Paila, identified and selected key stakeholders and health care providers based on their relevant work experiences and involvement in the development and/or implementation of interventions for women experiencing violence. Women seeking for regular health care services were approached by the study investigators, and those willing to participate in the study were interviewed. A total of six women with a history of violence were included in the focus group discussion (FGD).

### Participant recruitment

For indepth interviews (IDIs) and key informant interviews (KII): Health care centres were informed about the study via telephone or e-mail. Interviews were scheduled by setting a date and time, and available health care providers during our visit were interviewed using an interview guide. Women visiting health care facilities for general health or maternal and child health services at the time of our visit were invited to participate in an interview. Similarly, stakeholders from different local organizations who are actively engaged in violence prevention and advocacy were interviewed using an interview guide.

For FGD: Women with a history of violence were recruited with the assistance of a local woman who had herself been a victim of intimate partner violence. First, with the help of a local NGO, we identified and approached a local woman from the same community where the FGD was planned. This woman had a good relationship with women in the community. After explaining about the purpose of the study and her role as a participant, she was requested to recruit six other women in her neighborhood whom she knew had experienced violence to participate in the FGD. This approach allowed us to quickly identify the violence victims without making them retell their traumatic stories to the study team.

FGD with violence victims provided an opportunity for them to share their experiences regarding coping with and addressing IPV in their community, barriers to accessing VAW-related support services, and their recommendations for service provisions to better support the health and social needs of victims. Furthermore, interviews with stakeholders and women in general expanded our knowledge of the nature and consequences of violence, identified gaps in existing approaches to address violence and provided recommendations for future interventions/programs.

### Data collection

Qualitative data were collected through IDIs, KII, and FGD from January to February 2020 by trained interviewers. Data collection was stopped when no new information was forthcoming, indicating saturation of information [20]. Qualitative interviews and FGD were conducted in the Nepali language and audio recorded.

#### IDIs and KIIs:

The authors (RS, RRS) and two field staff members conducted all individual interviews, following a structured interview guide. The interview guide was developed after an extensive literature review and rigorous discussions among the research team and some key stakeholders working in the field of VAW in Nepal. Interviewees were asked about their perceptions of VAW, the measures that were being implemented in their setting to address VAW, what they believe is effective or not effective, and their ideas on how to effectively address VAW. Each interview lasted on average for 30 – 40 min. KIIs were conducted at the participant’s preferred time and place, while IDIs were conducted within health care centers in a confidential setting without any interruptions or disturbances. Unlike the general norm, women preferred to remain in pairs with their close friends during the interviews. As the interview questions did not include inquiries about their personal experiences regarding VAW, having two women in an interview was not an issue.

#### FGD:

One FGD was conducted with six local women residing in Baghai, a small village in the southern part of the Parsa district. This village was purposively selected based on information from a local non governmental organization indicating a higher prevalence rate of VAW in the area. Additionally, an additional study conducted in the same locale supports the assertion made by the local non-governmental organization regarding the high prevalence of violence against women in Baghai village [21]. The study revealed that domestic violence against women is a pervasive issue, with its underlying causes stemming from discriminatory social, cultural, economic, religious, and political customs and beliefs [21]. Based on these compelling findings and with the support of the local NGO, including women who have experienced violence firsthand, it was decided to conduct an FGD within Baghai village.

Similarly, FGD participants were informed by the FGD moderator about the aim of the study and their role and right to refuse or withdraw from the discussion at any time. All six women were from different households and married. A representative from a local NGO moderated the discussion, while two female members from the study team observed and took notes. The FGD was completed in approximately 60 min.

**Table 1:** Summary of study participants and data collection methods

S.N.	Participants	Method	Number of Interviews
1.	Health care providers (nurses and Auxiliary Nurse Midwives)	Semi-Structured Interview	15
2.	Women seeking general or maternal and child health services at health care facilities	Semi-Structured Interview	3
3.	Key stakeholder (local NGOs representatives who are working in the field of prevention of VAW)	Semi-Structured Interview	3
4.	Community women with a history of violence	Focus Group Discussion	1
**Total**			**22**

To ensure a comprehensive understanding of the phenomenon and capture diverse perspectives of the data, we employed a triangulation of methods, utilizing various data collection techniques such as FGD, KIIs, and IDIs. Furthermore, we incorporated multiple researchers at different levels, ranging from regional to national and local, to contribute to the data processing and analysis stages.

### Data processing and analysis

All interviewers underwent an intensive 3-day training program that specifically addressed qualitative methodologies, data collection tools, research ethics, and communication protocols, thereby fostering a shared understanding among them. The training was led by distinguished experts in the fields of qualitative research and public health. Transcription and translation services were provided by the proficient staff of Knowledge to Action (K2A), who were entirely independent from both the interview process and the study itself. Subsequently, the transcribed and translated data underwent meticulous scrutiny by language experts to ensure the utmost accuracy and validity. The interviews were first transcribed verbatim in Nepali and then translated into English. The transcripts were reviewed line by line and validated with audio recordings to maintain accuracy. The notes taken during the FGD were cross-checked by the moderator to ensure accuracy.

The data were analysed using qualitative content analysis. First, open coding was performed by independent researchers, and these codes were discussed within the study team. Second, the agreed codes were grouped into categories and themes. The themes were decided, and developed, and finalized based on the study objectives. However, any new and relevant themes emerging from the data were also added. Finally, these codes and themes were cross-examined, reviewed, and finalized after an agreement among the independent coders. The entire data analysis process was guided and carried out under the supervision of the research leader.

### Ethical consideration

The study received approval from the Nepal Health Research Council (NHRC: ethical review board number- 852/2019) and adhered to the World Health Organization’s (WHO) guidelines for ethical and safety recommendations for research on VAW [22]. All participants, including women with a history of IPV, who were invited to participate in the study, were provided with comprehensive information about the nature, scope, purpose of the study, potential risks and benefits. They were given the opportunity to have any questions addressed before providing their consent. Participation in the study was entirely voluntary, and participants had the right to withdraw from the study at any time, or to decline participation in any activities that made them feel uncomfortable, without the need to disclose their reasons. Written informed consent was obtained prior to enrollment. Participants were informed that the interviews would be audio-recorded. All the interviews were conducted in a confidential and secure space with a minimal chance of interference, such as a private closed room or secluded spot at the health facility. The tape-recorded and transcribed interviews were kept confidential in a locked cabinet, and no personally identifying information was collected. Participants were regularly reminded that the information they provided would remain confidential, and individual-level information would not be shared with anyone outside the research team. To minimize underreporting, experienced female interviewers were deployed and extensively trained on the interview guide. Participants were reimbursed with 500 Nepalese rupees as compensation for their time at the end of the interview or discussion. Some of the health care providers who participated in the study declined the incentive, stating that they were happy to share their experiences on such an important public health issue.

It was possible that women participating in FGDs might find it distressing to discuss their experiences of violence. This risk was mitigated by the use of trained female research staff for data collection, who were supported by research investigators (KD, RS, DS) with extensive experience of working with vulnerable women, including victims of IPV. Research staff were trained to refer women requesting assistance to available local services and sources of support. Furthermore, throughout the study, women participants were provided with an opportunity to seek support, either remotely or in-person, from a trained psychiatrist, who was a member of the research team. In adherence to the WHO guidelines on conducting research on violence against women [22], we collected information on the available support mechanisms in the district or province and developed a referral list that included the contact details of locally available IPV support services, such as crisis centers, hospitals, shelter homes, and community-based organizations, along with the respective contact persons. Women participating in FGDs were provided with this referral list, and researchers facilitated access to support services for those who needed or preferred additional support. We had also allocated resources to support women who expressed a need for counseling and support services. Conducting research on such a sensitive topic may cause emotional upset, stress, and anxiety for the researchers as well. To address this, regular monthly meetings among the research team were conducted, allowing research staff to debrief and receive support in managing their distress.

## Results

The majority of the health care providers interviewed belonged to the 16–35 age group and have completed a Certificate level in Nursing (Table 2). However, it should be noted that Table 2 does not include the sociodemographic characteristics of the community women and violence surviviors since we did not collect that information. Table 2 presents a concise summary outlining the sociodemographic characteristics of the participants.

**Table 2:** Sociodemographic characteristics of the participants

Variables	Number (15)
**Age**	
16–25	5
26–35	7
36–45	2
46–55	1
**Educational Status**	
University	3
Proficiency Certificate Level in Nursing	12
**Marital Status**	
Married	8
Unmarried	7
**Caste**	
Janjati	6
Brahmin	5
Vaisya	4

Triangulation and field observations of the qualitative data resulted in six themes: “Common Experience in Women’s Lives”, “Social, Cultural, and Traditional Norms Promoting VAW”, “Illiteracy”, “Underlying Reasons for Not Seeking Help”, “Health Center as the Primary Contact for Identifying and Managing VAW” and “Lack of Follow-up or Ongoing Support for the Survivors of Violence within Health Care Settings”. These themes are explained in detail below:

### Common experiences in women’s lives

All interview participants reported that women are forced to live with mental and physical injuries, which makes them afraid to break the silence. Some common physical impacts include injuries (ranging from minimal tissue damage to broken bones), pain, and impairment. Mental health impacts include stress, tension, fear, and even suicide. Intimate partner violence not only affects the individual but also the children and family of the violence survivors.

“Physical violence fades away along with healing wounds, but the effects of psychological violence are fatal and long-lasting in people’s minds and hearts, leaving deep scars. I think these mental tortures also create circumstances for a victim to commit suicide.”

KII participant (local NGO representative)

In addition to the effects of intimate partner violence on the individual, participants emphasized that it also has a significant impact on children’s health and well-being.

“If a husband does not care, the children will suffer. We will not be able to educate and feed (our) children properly.”

IDI participant (woman seeking health services)

“Violence is very contagious. It spreads from person to person and home to home. It has negative impact on the children. We further lose our prestige in the society.”

FGD participant (violence survivor women)

### Social, cultural, and traditional norms promoting VAW

Some common social, cultural, and traditional norms, such as son preference, dowry, and alcohol abuse, were found to be major contributors to VAW in Madhesh Province.

### (a) Son preference

Health care providers have identified “son preference” as a cause of VAW. Sons are considered to have greater economic and social value than daughters for various reasons. Firstly, daughters are seen as a financial burden and liability, mainly due to the expectation of future dowries. Secondly, sons are believed to carry on the family lineage, while daughters typically go to the groom’s house after marriage. Thirdly, since the government’s social security system for retired or elderly people in Nepal is insufficient, it is believed that sons will take care of their parents when they are old. Thus, sons are seen as the only financial security for elderly couples in the absence of a social security system. Lastly, there is a traditional norm that the son should perform the last ritual for a deceased parent, further reinforcing son preference. The longstanding social and traditional norms often lead couples to repeatedly conceive until they have a son, and women are often blamed for not giving birth to a son. Women who fail to give birth to a boy experiences a loss of respect within the family and are frequently subjected to physical and verbal abuse, threats, and sometimes even forced eviction. In some cases, husbands may even marry another woman in the hopes of having a son.

“If there are 4–5 daughters, violence against the women begins. Family members say, “You always give birth to daughters, not sons”. They use derogatory terms such as ‘Hijdi’ (which translates to ‘transgender’). The in-laws scold her, saying, “You do not have a son, so you are a sinner.” Nobody blames the son, but they blame the daughter-in-law. She might even commit suicide, as she feels she has nowhere to go.”

IDI participant (Health care provider)

“There was a case where a woman was in her third pregnancy (Gravida 3). She had two daughters before. Her husband used to beat her for giving birth to daughters due to pressure from his family, especially when he had been drinking alcohol. He threatened to abandon her or throw her out of the house if she did not give birth to a son this time.”

IDI participant (Health care provider)

### (b) Alcohol abuse

Most of the participants including all FGD participants, six health care providers, and two women seeking health services, identified alcohol abuse by husbands as one of the reasons for VAW. When husband are drunk, they instigate fights for no reason, verbally abusing women, often resulting in physical injuries. Apart from physical violence, women also experience psychological distress due to fear, stress, and worry about being beaten and the consequences that may unfold when their husbands return home.

“If we do something wrong, they beat us. If we say something, they beat us when they are drunk” (said in a low tone, accompanied by slight laughter).

FGD participant (Violence survivor woman)

“Some instances of violence occur when husbands drink alcohol. When they are drunk, they neglect their wives and children and subject them to abuse.”

IDI participant (Woman seeking health services)

“One of the reasons for wife-beating is alcohol consumption. When husbands come home drunk, they abuse their wives, and if a woman tries to say something, he physically abuses her.”

IDI participant (Health care provider)

### (c) Dowry

Most of the participants from all categories (including all FGD participants, two women seeking health services, and eight health care providers) identified dowry as one of the contributing factors to VAW in Madhesh Province. If the bride’s family is unable to meet the dowry demands of the groom’s family before or after marriage, the woman may be subjected to torture, abuse, and taunting to obtain the dowry from her natal parents. Two health care providers mentioned encountering cases of miscarriage and physical injuries resulting from dowry issues. One of the survivors of intimate partner violence recounted being abused and sent back to her maiden home for a year until the dowry demand was fulfilled.

“One of the reasons (for violence) is the dowry system; community people start giving advice such as, ‘If you marry another woman, you will receive certain property, so leave the current one.”

IDI participant (Woman seeking health services)

“I was sent back to my natal house by my in-laws because my parents could not provide the promised dowry within the three months of marriage. They (in-laws) said that they would only take me back only when my parents provided the remaining dowry amount as agreed upon. I later returned to my husband’s home after my father fulfilled the dowry requirement.”

FGD participant (Violence survivor woman)

“Dowry is another reason. Women are subjected to physical assaults and are not allowed to live in the house. Husbands have extramarital affairs and insult their wives when they are unable to provide the demanded dowry.”

IDI participant (Health care provider)

In contrast, one health care provider mentioned that violence due to dowry is less prevalent, as most people nowadays are educated, and violence has decreased over time.

### Illiteracy

Participants, excluding women seeking health care services, pointed out that a low level of education contributes to VAW. Different interviewee categories held different views regarding the level of education of perpetrators or women as a contributing factor to VAW. Violence survivors believed that a low level of education among perpetrators contributes to VAW. They believed that educated people are more understanding and would prefer to communicate resolve issues rather than resorting to violence. Additionally, they believed that if men were more educated, they would consume less alcohol. However, health care providers and representatives from local NGOs perceived that the low level of education among women leads to VAW. They believed that if women are more aware of their rights and educated, they would be empowered to report VAW to the authorities, seek legal and social assistance, and prevent further victimization of the survivor.

“In my point of view, the lower caste people (uncomfortable using this term) who have received limited education tend to be more violent, but educated people can understand and know how to mend things.”

IDI participant (Health care provider)

“Uneducated people who have not received formal education may resort to poisoning, murder, hanging, or burning their wives with petrol or kerosene.”

IDI participant (Health care provider)

“Every man abuses his wife. Its just that since we are not educated, violence is even more common. If he (the husband) says something and we interfere in their work, they start abusing us. If the food is not tasty, then they beat us.”

FGD participant (Violence survivor women)

### Underlying reasons for not seeking help

A small number of health care providers (4 out of 15) stated that most or all victimized women seek help. Only a few participants (2 out of 15) mentioned concerns about fake allegations and believed that most violence survivors would choose to discuss the violence they are experiencing with someone.

“The real victims who are suffering from this stuff [violence] openly share their experiences they face with a health care provider and their family/friends.”

IDI participant (Health care provider)

In contrast, the majority of the health care providers (9 out of 15), all violence survivors, and representatives from local NGOs expressed concern that survivors do not seek help from formal and informal support systems. Women are forced to live with mental and physical injuries as they are afraid to break the silence, resulting in unreported cases. Some commonly reported reasons for not seeking help are grouped under the following subthemes.

### (i) VAW is regarded as a normal family matter

Acceptance or normalization of violence against women in the community is a major barrier to seeking help or assistance. We found that all FGD participants, one health care provider, and one key informant believed that domestic and/or intimate partner violence is considered a normal family matter and not perceived as a problem that needs to be addressed or managed, nor is it recognized as a violation of human rights. The casual expression of existing VAW in the community by a participant quoted below also indicates the deep-rooted acceptance of VAW in society.

No one fights all the time, but violence must have occurred in 98% of women living in this locality (states very casually).“

FGD participant (Violence survivor woman)

All violence survivors accept violence as the fate of women and something that is inevitable. They believe that husbands have the right to exert control over them, and that slaps and verbal abuse are common in a marital relationship. Women only report intimate partner violence to their natal family when it becomes severe.

“We have to face[violence], all that is written in our fate.“

FGD participant (Violence survivor woman)

“All women whole-heartedly accept their partners and never complain about anything as this is our culture. We often hear women saying, “He is my husband, and he has the right to slap me or beat me. The husband makes every decision in a family; it is no big deal for us.”

KII participant (local NGO representative)

“Many women do not want to disclose that their husband or in-laws are torturing them. They only speak up if it becomes unbearable. They don’t openly talk about it due to fear that their husband and in-laws might kick them out of the house. We are also women and live in the same house with our spouses and in-laws. Our husbands say many things as well, but women have to endure such things.”

IDI participant (Health care provider)

### (ii) Silence due to fear of escalating violence

Many women prefer to remain silent out of fear that the violence may escalate once the perpetrator becomes aware of the disclosure of the violence experience to others.

“It is due to fear that her husband and mother-in-law might evict her from the house.”

IDI participant (Woman seeking health services)

“Women here do not go out and seek help for their IPV experience. He (husband) is our own man; he beats and slaps us, but we do not separate. Women here are scared of their husbands”.

FGD participant (Violence survivor women)

“Men warn us not to seek help or go anywhere for support because the violence will increase even more. So, where can we go? We remain silent and stay at home.”

IDI participant (Women seeking health services).

The uncertainty of the consequences faced by the perpetrator if charged by the police or the duration of their incarceration, dependency on the perpetrator due to limited economic empowerment, and the fear of worse consequences upon returning home also deter women from seeking help from formal sectors, such as the police or judicial system. Victimized women become more reluctant to discuss violence in health facilities if other family members are present.

“A victim does not want to conceal it, but their in-laws and sisters-in-law, try to hide it. We do not allow visitors go inside the labor room, so when we ask them at that time, and they open up to us.”

IDI participant (Health care provider)

This fear can also stem from a lack of trust and confidentiality among health care providers. Women fear that sharing their problems with health care providers may result in the dissemination of this information, which would alert the perpetrator and put the woman would be at an increased risk of future victimization.

Women who have experienced intimate partner violence and shared their concerns with health care providers stated, “What if, my husband or family members beat me later after I have shared this? They might even kill me. After all, they have threatened me not to share about these things. It will spread throughout the community.”

IDI participant (Health care provider)

Health care providers should allocate sufficient time and effort to develop trust and rapport, as these are essential in helping survivors disclose IPV.

“They do not spontaneously open up about everything; it takes a lot of time. We dedicate a minimum of 2–3 hours and of 4 hours for one case.”

IDI participant (Health care provider)

Women seeking health services fear that involving the police would tarnish their family’s prestige, as the whole society would become aware of it. They dread the judgements that people might pass. Likewise, involving the police in cases of violence is not seen as a long-term solution. The perpetrator is eventually released, and there is a fear that the violence will escalate even further. Since most women are economically dependent on their husbands for their livelihood, they feel reluctant to take any action against them. Despite the existence of limited safe housing options, victimized women do not seek help or choose not to enter safe housing.

“Even if I try to defend myself or we have them beaten once, they ultimately come back home (uneasy laughing).”

IDI participant (Woman seeking health services)

### (iii) Social stigma surrounding IPV

Biased gender norms lead family members to believe that it is a woman’s responsibility to care for her husband’s family and fulfill the needs of her own family. If she fails to meets the expectations, she is deemed a bad daughter-in-law. This societal notion of an ideal daughter-in-law contributes to women’s reluctance to seek help. Survivors of violence express concerns about how society will perceive them if they disclose their experiences of violence.

“I understand why women hesitate to come forward. They fear acceptance by society. What will people say? Will they love me if I return to my parents’ home, or will they humiliate me?, etc. such things are usually hidden within families in villages due to fear.”

IDI participant (Health care provider)

“We go to our maternal house and stay there in extreme situations so that rumours do not spread.”

FGD participant (Violence survivor woman)

Health care providers and representatives from local NGOs also point out that women face numerous socially imposed restrictions. For instance, they are prohibited from interacting with strangers, are expected to adhere to certain behaviours, and speech patterns, and are restricted in their choice of clothing. These limitations constrain women and confine them within narrow boundaries. Because women are constantly under societal scrutiny, they are afraid to speak up.

“If a female goes from one house to another, then people gossip about her, saying things like ‘so and so’s daughter-in-law went to such a place.’ There is no freedom for women here.”

IDI participant (Health care provider)

### Health center as the primary contact for indentifying and managing VAW

Women with evident physical bruises and injuries resulting from different physical violence often seek help at outpatient departments or emergency units to receive treatment. Nurses often identify individuals as survivors of violence during history-taking, but this seldom or rarely leads to a police report or any social interventions. They address apparent physical issues such as bruises, burns, and other injuries, and provide counseling to the women, often offering words of encouragement. However, they rarely advise the woman to leave their perpetrator. Five health care providers were trained in psychosocial counseling or in how to handle violence-related issues.

“First of all, we treat the wounds if they (victimized women) come up with it. After managing the wounds, we asked them about how the incident occurred. We try to counsel them on how to minimize such incidents. We counsel them according to our knowledge.”

IDI participant (Health care provider)

Health care providers also mention cases where a patient expriences extreme fear and tension, indicating that survivors often endure psychological violence alongside physical violence. However, these cases are often not reported unless they manifest as physical or sexual violence. Four health care providers also mentioned that they seldom encounter cases of sexual violence that escalate to rape, as rapes are reported less frequently.

However, three survivors of violence and women seeking health services had a different take-on violence management at health care centers. They raised concerns about the ability of health care centers or other formal institutions to reduce or prevent violence. They prefer to seek help from community leaders or elderly people within families over health care centers to settle disputes, as they are highly regarded and respected in the community.

“People here do not have faith in health centres. Although health centres provide services, they are not sufficient to reduce violence. When it comes to discussing our feelings, we prefer Mukhiya (traditional leaders and decision-makers in villages/communities) and the ward chairman, they are sought to settle down disputes and minor conflicts rising in the village/community.“

FGD participant (Women survivor of IPV)

“If anyone comes to the health center with a complaint of violence, they do not pay any attention to it. They would refer the person to Birgunj (a metropolitan city in the Parsa district of Madhesh province). However, people here are very poor and cannot afford the hotel bills, so where would they stay in Birgunj? There is a problem here.”

IDI participant (Women seeking health services)

### Lack of follow-up or ongoing support for the survivors of violence within health care settings

There is no standard procedure or protocol in the health care centers regarding when and how to follow-up with survivors of violence. Health care providers mentioned that due to the lack of follow-up and ongoing support for identified survivors, they remained unaware of the survivor’s situation after the initial counselling.

“We provide our recommendations, but they do not come for follow up. I mean, these types of cases do not show up for follow-up, so we do not have much information about them. However, we offer counselling from our side, based on our knowledge.”

IDI participant (Health care provider)

Service providers were unaware of the effectiveness of their counselling services since they had not undergone formal evaluation.

“They nod and say yes, but we do not know what they would do after going back home. They appear calm and relaxed during counselling. Victimized women show genuine interest in taking action, but we have no idea if they implement those changes in their lives.”

IDI participant (Health care provider)

### Recommendations for reducing/preventing violence

Participants suggested different measures and strategies for reducing or preventing violence. One of the most common suggestions from participants was awareness-raising programs that provide information about the nature and impact of violence. These programs should also include information about the support services offered by the government and NGOs. Awareness can be raised through various communication channels.

“Awareness programs about One-Stop Crisis Management Centers are necessary because most of the people are unaware of their purpose and function? The public lacks knowledge about it. If people know that privacy is maintained here, they will be more willing to seek help.”

IDI participant (Health care provider)

Participants, especially nurses, strongly believed that counselling would benefit survivors. Counselling should not only be limited to survivors but should also be extended to husbands and families, as this can help reduce violence. Participants emphasized the importance of support from male members of society for the success of any intervention.

“First, men should understand the consequences of intimate partner violence and how the patriarchal mindset contributes to gender discrimination in families and communities. Men should know women’s rights. Men should support their wives in engaging in income-generating activities. They should understand the multiple roles women have in household work and respect women’s unpaid work.”

KII participant (local NGO representative)

Apart from this, the study participants also highlighted several measures, such as strict enforcement of laws, informing women about the rules and legal matters, encouraging women to seek help, banning alcohol and stopping the dowry culture. These measures would significantly contribute to preventing violence.

“When a man comes home after drinking alcohol, he scolds; female also yells back at the man. They scold each other and end up fighting. If alcohol is banned, it would lead to improvement among all-men in the village.”

FGD participant (Violence survivor woman)

Survivors of intimate partner violence emphasize the importance of education and employment opportunities for men as preventive measures. Improved education empowers men, reduces alcohol consumption, and contributes to changing socio-cultural practices that promote VAW.

“If there are employment opportunities, men will engage in them, and the violence will not occur. If they have job, they won’t have as much free time. Staying idle causes violence. Men do violence if they have more spare time.”

FGD participant (Violence survivor woman)

Representatives from local NGOs and health care providers recommended improving education and employment opportunities for women to prevent violence. Education empowers women and shifts the help-seeking behaviour from traditional community leaders to formal institutions such as police. Employment opportunities will make women financially independent enabling them to leave their perpetrators.

“There is a saying that if education is given to women then they automatically empower themselves but more than just education, women need empowerment through different means and training.”

KII participant (local NGO representative)

### Perception regarding counselling intervention

Health care providers and representatives from local NGOs believe that hospital-based counselling interventions could be effective for women who have experienced violence. Since health facilities are often the first place that severely injured women go to, it can be a good starting point to initiate programs addressing mental health problems related to intimate partner violence or domestic violence. It also provides an opportunity for women to openly share their experiences with health care providers.

“I think hospital-based counseling intervention is effective because women (clients/patients) feel free in the hospital, and can share their experiences with medical person about their intimate partner [and violence].”

KII participant (local NGO representative)

“I believe counseling is highly effective because, when a person cannot open up with their relatives or anyone they know, they are unable to share everything. They are also in fear that the information might spread. However, in this place (hospital), they can share it easily with an unknown person (health care providers). They will get the platform to bring out all the things they have faced and seek help. I think it is effective and reduces mental stress in the long run.”

IDI participant (Health care provider)

### Need of training health care providers

In-depth interviews with the health care providers highlighted the importance of training for dealing with women experiencing violence.

“I think it would be easier if we had a counselor who could provide counselling to women experiencing violence here. We can give them to handle these (violence) cases. I am the only person who has been trained in this area. If we could provide training to other staff as well, they could work even in my absence.”

IDI participant (Health care provider)

“At present, we do not offer counseling services to women experiencing violence because we have not received any training so far. But if we receive training for counseling such cases, then we will provide it. We cannot say that we will not help them. It is a necessity for everyone.”

IDI participant (Health care provider)

“If there is any training that is better than the previous one, then it would be better if you could provide it to us. We only know how to handle them until now. If there is better training available, we would be happy to receive it. I believe it will enhance our ability to manage women experiencing violence.”

IDI participant (Health care provider)

## Discussion

This qualitative study aimed to explore the perceptions of VAW among health care providers, women visiting health care settings, VAW survivors, and relevant stakeholders in Madhesh Province in Nepal. This study was conducted as part of a baseline assessment preceding a planned intervention to address VAW and its mental health consequences by mobilizing health care providers.

The participants identified several key issues in Nepalese society that exacerbate and perpetuate the risk of VAW. These include social and traditional norms that support a patriarchal society, such as dowry, son preference, silent tolerance of taboos, overconsumption of alcohol, low levels of education, and lack of law enforcement. These findings align with studies conducted in areas other than Madhesh province of Nepal [17, 23].

Similar to previous evidence, son preference emerged as one of the primary reasons for violence against women [8, 17]. Son preference is prevalent in patriarchal societies like Nepal [24], especially among the Madhesi community, which refers to the people living in Madhesh, the plain southern belt of Nepal. Sons are preferred in most Nepalese families due to religious, social, traditional, and cultural values. Sons are seen as a source of income for the family, a means to maintain the family lineage, and are necessary for performing funeral rituals [17, 25]. This bias in preference towards sons also indicates the unequal treatment daughters receive from an early stage in childhood. Not having a son also often leads to separation and polygamy [8]. A qualitative study conducted in the Kapilvastu and Rupandehi districts of Nepal demonstrated that norms and cultural practices, such as the Gauna system (a practice where children are married at a very young age, typically between 8 and 14, but only move to the husband’s house several years later, usually at age 17 or 19, after the ‘Gauna’ ceremony takes place), marrying the wife of a deceased brother, and staying at the natal home during childbirth, also contribute to violence [26].

Although the Gauna system was not a specific finding of our study, we found that other cultural norms, such as the dowry system, were significant contributors to violence. The practice of dowry remains prevalent, especially among the Madhesi community, and has been identified as an important factor in initiating or escalating violence against women. Dowry is also practiced in other countries like India and Bangladesh, where studies have demonstrated its association with violence [8, 27]. The government of Nepal has taken the initiative to eradicate the dowry system and strongly condemns the practice. The Penal Code (2017) includes provisions for a fine of up to 30,000 Nepalese Rupees and imprisonment for three years for those involved in dowry transactions [28, 29]. While the prevalence of the dowry culture has decreased, but it has not yet been completely eliminated. Evidence of its persistence can be found in newspapers and police reports [4].

These prevailing patriarchal norms hinder women from seeking help. Research conducted in Nepal reveals that women refrain from reporting instances of violence to concerned authorities or family members due to the fear of losing their livelihoods, social stigma, embarrassment, and the risk of further increase of violence [12, 30]. Additionally, violence is perceived as a private matter that should not to be discussed [18]. A study carried out in Pakistan demonstrated that violence is deeply rooted in family beliefs and cultural practices [31]. The authority of men over women and the cultural, social, and traditional constructs and characteristics are so ingrained in Nepali society that women are constantly expected and obligated to silently endure their suffering. Women are taught to endure all pain and sacrifice for the common well-being of the family [8]. Patriarchal masculinities encompass financial responsibility for the family, displaying leadership, suppressing emotions, making household decisions, and being perpetrators rather than victims of violence. However, women’s traditional roles are limited to household chores and childbearing. The strength of masculinity and the presence of unequal gender norms disempower women from fighting for their rights, which leaves men in a positions of power. This lack of agency, voice, and position within the household also contributes to intimate partner violence and discourages survivors from seeking help. Another study conducted in a different region of Nepal compared marital conflicts to a “hay stick fire”, implying that the dispute ignite and extinguish easily, and that couple will eventually reconcile, making interference seems unnecessary [8]. This not only normalizes violence but also deters others from offering assistance.

Another recurrent issue identified in the study was the low level of education, which diminishes awareness among both survivors and perpetrators that VAW does not have to be an accepted way of life, violating human rights and laws intended to prevent VAW and child marriage. The lack of education and empowerment further restricts women’s opportunities to work outside the home and reduces families’ dependence on dowries. Alcohol consumption was frequently cited as a significant factor contributing to VAW.

Evidence from a study indicates that women prefer being asked about their experiences by health care providers [12]. However, some of the participants in our study did not favor the idea of opening up to health care providers. Women may have been skeptical about seeking help from the health care providers, fearing potential humiliation, embarrassment, and a breach of confidentiality. Health care providers often fail to inquire about violence as part of routine assessments [18]. Providers believe that women would be more forthcoming during counselling once assured of confidentiality. Additionally, there is scientific evidence supporting the benefits of individual counselling over group trauma-focused interventions in providing mental health support to survivors of violence. Individual counselling has shown long-term reduction in anxiety and depression, lower attrition rates and the therapeutic relationship is considered more impactful than the social support provided in group settings, which tends to fade over time [32]. Similar studies conducted in India also provide evidence that formal interventions such as individual and family counselling lead to improved coping strategies and reduced stress among victims of violence [33].

The necessity for increased training on mental health management for Nepalese health care providers is supported by another study conducted in the Chitwan district of Nepal [34]. Studies focusing on pregnant women have demonstrated that a nonjudgmental approach towards women would facilitate willingness to seek help [12, 35]. Although several government health facilities offer One-Stop Crisis Management Centres, many women are unaware of these services, and those who are aware tend to visit these centres only in severe cases of violence. There is a weak functioning system in place for health or social workers to assist women in filing charges with the police or providing safe housing for female survivors and their children in Nepal.

While the country has robust policies concerning women, these policies are not effectively enforced in practice due to a lack of implementation mechanisms. The Nepal Police has initiated and established the “Women, Children, and Senior Citizenship Services”, also known as “Women’s Cell,” with the primary objective of ensuring women’s safety in cases of committed violence against them [36]. Despite the protection and fair treatment services offered by the Nepalese police, the reporting and seeking of legal support are minimal due to several factors: the assumption among abused women that physical violence is an acceptable means of conflict resolution in relationships, fear of retaliation from close perpetrators, economic dependency, embarrassment, lack of awareness about the signs of abuse and the preventability of violence. Stigma, shame, taboo, and the fear of ridicule also lead some abused women refrained from reporting. Additionally, there is a lack of trust among women in national and state authorities [30] when it comes to enforcing the laws and policies for survivors. Similar findings were observed in a study conducted in India, where victimized women avoided seeking help in order to maintain family integrity, fearing further abuse, and believing that situation would not improve [27].

### Proposed strategies for addressing violence

The study findings suggest that the majority of women who experience violence do not seek health services or any kind of support for violence prevention. They do not believe that health facilities will help in violence prevention, and concerns about privacy infringement prevent them from seeking help. On the other hand, health care providers stated that once the women were aware and convinced regarding confidentiality issues, they opened up. Below are some recommded strategies to address the issue of violence:

### (a) Public awareness

Women experiencing violence are often hesitant to seek help or support from both formal and informal mechanisms. This reluctance stems from perceiving VAW as a normal family matter that may resolve on its own over time, as well as from the fear of violence escalating and the widespread social stigma associated with VAW. Thus, it is essential to raise awareness about the reasons behind violence, its impact on women, families, and communities, and the fact that VAW is a criminal offence. Increasing awareness of existing laws and policies against VAW is also crucial.

The preference for sons is deeply ingrained in patriarchal societies like Nepal and primarily stems from poor awareness that the biological sex of a child is beyond the influence of the mother. It is important to raise awareness of how a child’s sex is determined using local television channels, radios, mothers’ groups, local clubs, or social media. These efforts can help eliminate the blame on women for not giving birth to a son. Trusted people in the community, such as community leaders, health care providers, and teachers, should be included in planning, designing, and implementing local advocacy campaigns. As VAW is a medical, social, and human rights problem, awareness programs should target couples, families, and the entire community.

### (b) Sensitize and train health care providers on violence management

Health care providers are in a crucial position to identify survivors and provide the necessary help. The current study shows that adopting a proper and secure approach (creating a supportive environment, assuring confidentiality, and respecting their decisions, opinions, values, and choices) would enable effective management of VAW and its consequences. In the current scenario, health care providers may not have the required skills to deal with such a sensitive issue, but they are assisting survivors to the best of their knowledge. Therefore, sensitizing health care providers in dealing with VAW and its mental health consequences could be a crucial step in managing violence survivors in the low-resource setting of Nepal. Health care providers should be trained in rapport building, approaching and screening women experiencing violence, and guiding and supporting victimized women. Similarly, a recent qualitative study from Nepal [37] highlighted components of training. This training should not be limited to the One-Stop Crisis Management Centre sites but should be provided in all health facilities. Apart from counselling, health care providers can also contribute in other ways by educating the men about the problems of violence during their visits, such as informing them about the use of the contraception and the need for care during pregnancy.

### (c) An intervention involving community people/male members/husbands

Since there are multiple factors contributing to VAW, a multidimensional approach should be employed to address them. Our study revealed that women prefer seeking assistance from their family or community leaders rather than health centers, police, or any other concerning bodies. Therefore, any intervention aimed at addressing VAW should incorporate community components. Intervention programs should target the community and the family as a whole, enabling family members and/or the community to provide support to women in addressing VAW.

### (d) Strict enforcement of laws and regulations

To support favorable environment for survivors to seek help, laws and policies regarding VAW should be strictly implemented. Implementation of the law should establish a conducive environment that rebuilds trust and faith in seeking justice and instills fear among perpetrators, deterring them from committing acts of violence. Sensitizing and training police personnel working in the local communities about VAW and its consequences can help initiate VAW-related law enforcement and reduce dowry or VAW cases that are resolved outside the court.

### Strengths and limitations

This study stands out as one of the few studies that has explored the perceptions of diverse groups of individuals, including survivors of violence, health care providers involved in the health care of survivors, women seeking health care services, and relevant representatives from local NGOs working on violence prevention, its causes, and its impacts on communities. Moreover, the study has large geographic coverage, as the data were collected from eight districts. The exploratory approach of the study has yielded in-depth information that has made significant contributions to the design and implementation of a planned multicomponent intervention, which will soon undergo testing through a cluster randomized trial.

One limitation of the study was the lack of detailed information on sexual violence, as participants exhibited greater reluctance to discuss the topic. This finding aligns with other exploratory studies on domestic violence in rural Nepal [38, 39]. Due to its classification as a private matter, most participants felt hesitant and ashamed to disclose incidents of sexual violence. In Nepal, forced sexual intercourse by a husband within marriage is not considered rape, as the prevailing belief is that once a woman is married, her husband has the right to engage in sexual relations without her consent. The Nepalese criminal code, adopted in 2017, defines rape as “sexual relations with a woman without her consent and with a girl under the age of 18 with her consent”. Regarding marital rape, the criminal code stipulates that “if a man rapes his wife while still in a marital relationship, he can be sentenced to up to 5 years in jail” [40]. Additionally, women remain largely unaware of the legal provision that recognizes marital rape as a criminal offense. Qualitative studies conducted in Bangladesh and Finland have demonstrated that women who experience physical and sexual violence often exhibit higher levels of mental and psychological distress [41, 42]. Obtaining in-depth information on sexual violence requires conducting more one-to-one interviews with an information-rich study population.

Another limitation of the study relates to the translation of cross-cultural qualitative research. To ensure the essence of the interviews was retained during translation, efforts were made to translate local words and phrases exactly as the participants had responded. Nevertheless, the four co-authors were fluent in both languages (Nepali, English, and other local languages). During the translation process, the meaning of local words, slangs, and the interviewees’ perspectives were described in detail. This approach aimed to provide other authors who only read the English translation with an accurate representation of the actual scenario and assist in the analysis. Additionally, this study excluded the health posts, which raises the possibility that health workers in the health posts might hold different perceptions compared to health workers in primary health care and hospitals with One-Stop Crisis Management Centres. This factor has the potential to affect the generalizability of the study’s findings, particularly considering the diverse ethnicities and remote scattered villages present in the study area.

## Conclusion

Most women and stakeholders expressed that women in the community have experienced one or more forms of violence, with physical violence being the most prevalent, followed by psychological violence. Health care providers also reported instances of sexual violence, which often remained unreported. The study identifies sociocultural practices such as dowry, son preference, gender norms, gender power relations, and low awareness among community members regarding VAW and its consequences as major contributors to violence in Madhesh Province.

While healthcare interventions for supporting survivors of violence are emerging, they remain limited in scope, and most interventions have not adequately incorporated the perspectives of victims’ during their design. Additionally, survivors of violence reported that available healthcare interventions primarily focus on providing care and support for those with visible physical injuries. They also expressed concerns about the limited availability of trained counsellors or support personnel, as well as inconsistencies among health facilities in terms of services provided to victims.

Considering that health care providers play a crucial role in identifying and supporting survivors of violence, implementing counselling interventions by trained health care providers appears to be both feasible and potentially effective in addressing VAW. Given the multiple factors contributing to the initiation and/or escalation of violence, it is imperative to conduct trials and tests of a multicomponent support intervention delivered by trained health care providers.

## Data Availability

The datasets generated and/or analyzed during the current study are not publicly available due to other ongoing studies but are available from the corresponding author on reasonable request. After completion of all the planned studies of project, datasets will be available in the following weblink: https://knowledgetoactions.com/knowledgetoactions/.
